# The Role of Serum CK18, TIMP1, and MMP-9 Levels in Predicting R0 Resection in Patients with Gastric Cancer

**DOI:** 10.1155/2018/5604702

**Published:** 2018-02-05

**Authors:** Sevki Peduk, Cihad Tatar, Mursit Dincer, Bahri Ozer, Ahmet Kocakusak, Gamze Citlak, Muzaffer Akinci, Ishak Sefa Tuzun

**Affiliations:** ^1^Department of General Surgery, Haseki Training and Research Hospital, Istanbul, Turkey; ^2^Department of General Surgery, Istanbul Training and Research Hospital, Istanbul, Turkey; ^3^Department of General Surgery, Kemerburgaz University School of Medicine, Istanbul, Turkey

## Abstract

Gastric cancer is the third most common cause of death in men and the fifth common cause of death in women worldwide. Currently, available advanced imaging modalities can predict R0 resection in most patients in the perioperative period. The aim of this study is to determine the role of serum CK18, MMP-9, and TIMP1 levels in predicting R0 resection in patients with gastric cancer. Fifty consecutive patients scheduled for curative surgery with gastric adenocancer diagnosis between 2013 and 2015 were included in the study. One milliliter of blood was taken from the patients included in the study to examine CK18, MMP-9, and TIMP1. CK18, MMP-9, and TIMP1 levels were positively correlated with pathological N and the stage (*P* < 0.05). The CK18, MMP-9, and TIMP1 averages of those with positive clinical lymph nodes and those in clinical stage 3 were found to be higher than the averages of those with negative clinical lymph nodes and those in clinical stage 2 (*P* < 0.05). Although serum CK18, MMP-9, and TIMP1 preop measurements in patients scheduled for curative surgery due to gastric adenocarcinoma did not help to gain any idea of tumor resectability, we concluded that our study had valuable results in significantly predicting N3 stage.

## 1. Introduction

Cytokeratins are keratin-containing proteins of the family of intermediate filamentous filaments, found in the intracytoplasmic cytoskeleton of the epithelial tissue. CK18 is involved in important signaling pathways such as apoptosis, cell cycle, and cancer progression that regulate cell behavior. Especially with caspase-mediated cell death, the serum level is elevated. Matrix metalloproteinase-9 (MMP-9) disrupts the extracellular matrix and affects almost all aspects of cell biology. MMP-9 is one of the most complex matrix metalloproteinases. The tissue inhibitor of metalloproteinase-1 (TIMP1) plays a negative role in the invasion and metastasis of tumor cells by forming a 1 : 1 complex with MMP-9 and inhibiting its enzymatic activity [1–7]. Gastric cancer is the third most common cause of death in men and the fifth common cause of death in women worldwide [8]. Today, surgery is the only curative therapy for stomach cancer. One of the main determinants of survival is the type of surgical dissection done. Currently available advanced imaging modalities can predict R0 resection in most patients, but it can only be detected in the perioperative period, and some cases are unresectable. The aim of this study is to determine the role of serum CK18, MMP-9, and TIMP1 levels in predicting R0 resection in patients with gastric cancer.

## 2. Materials and Methods

In the General Surgery Clinic of the University of Health Sciences Haseki Training and Research Hospital, 50 consecutive patients scheduled for curative surgery with gastric adenocancer diagnosis between 2013 and 2015 were included in the study. Esophagogastroduodenoscopy was performed preoperatively, and endoscopic biopsy results were evaluated as adenocancer. Clinical staging of the patients was performed with IV contrast-enhanced thorax and whole abdominal CT. Patients thought to be inoperable and/or unresectable for clinical staging, patients with neoadjuvant treatment, and patients with a pathological diagnosis outside of adenocarcinoma were excluded from the study. Our study was conducted with the approval of the Ethical Committee for Non-Drug Clinical Researches dated January 1, 2016, and numbered 302. One day prior to the operation, 1 ml of blood was taken from the patients who were included in the study to study CK18, MMP-9, and TIMP1, which were taken for routine investigations and placed in separator gel-containing biochemical tubes. The blood was centrifuged at 10,000 rpm for 5 minutes and stored at −50°C.

The cost of kit purchased for CK18, MMP-9, and TIMP1 was covered by the Advisory Monitoring Committee of Theses and Academic Studies. All of the patients were informed about the study, and their written consents were obtained.

## 3. Statistical Analysis

SPSS 15.0 for Windows, Mann–Whitney *U* test, Kruskal-Wallis test, and Bonferroni correction were used for statistical analyses. Statistical significance level of alpha was accepted as *P* < 0.05.

## 4. Results

Patient characteristics are given in Table 1. Of the 50 patients studied, 31 (68%) were male and 19 (32%) were female, and the mean age was 64 (min-max: 42–88). 20 of the patients (40%) were ASA 1, and 30 (60%) were ASA 3. The mean gastric tumor size of the patients was 34.1 ± 6.7 mm (min-max: 21–47 mm). Six (12%) patients underwent palliative surgery, and 44 (88%) patients underwent R0 resection. Subtotal gastrectomy + D2 lymph node dissection was performed in 24 (48%) patients, and total gastrectomy + D2 lymph node dissection was performed in 20 (40%) patients. D2 lymph node dissection range varied between 14 and 24 nodes. 10 (20%) patients underwent additional organ resection, 2 of these patients (4%) had colon resection, 3 of them (6%) had cholecystectomy, and 5 of them (10%) had splenectomy. Seven (14%) of the patients had stage 2B, 6 (12%) had stage 4, and 37 (74%) patients had stage 3 stomach cancer. Average follow-up time was 21.28 (min-max: 1–40) months.

CK18, MMP-9, and TIMP1 levels were positively correlated with pathological N and the stage (*P* < 0.05) (Tables 2 and 3).

The CK18, MMP-9, and TIMP1 averages of those with positive clinical lymph nodes (pathological lymph nodes in preop tomography) and those in clinical stage 3 were found to be higher than the averages of those with negative clinical lymph nodes and those in clinical stage 2 (*P* < 0.05) (Table 4).

## 5. Discussion

It has been reported that CK18 may exhibit an abnormal expression in various cancers and may provide information about the prognosis. Makino et al. [9] reported that an increase in CK18 expression in esophageal squamous cell carcinoma was associated with poor prognosis. Messai et al. [10] reported about this association in renal cell carcinoma; and Fillies et al. [11] reported that an increase in the CK18 expression in oral cavity was correlated with a rise in stage and class. However, Knosel et al. [12] reported that low CK18 expression in colorectal cancer was associated with poor prognosis. Buhler and Schaller [13] emphasized that transfection of the CK18 gene in human breast cancer cells led to induction of adhesion proteins and dramatic retreat of *in vitro* and *in vivo* malignancies. In a study conducted on lung cancer patients by Zhang et al. [1], high CK18 expression was determined in 101 (78.3%) of 129 patients. CK18 expression correlated significantly with clinical stage, lymph node metastasis, number of pathologically positive lymph nodes, and recurrence and metastasis. Scott et al. [14] reported that the preoperative CK18 levels in patients with gastric adenocarcinoma and that a significant fall in these levels after treatment allowed the assessment of the tumor response to the treatment. Oyama et al. [15] mentioned the importance of the levels of caspase-dependent (M30) and total (M65) fractions in CK18 serum in predicting the prognostic factor in gastric adenocancer patients. Bilici [16] reported that CK18 fragments (M30, M65) could be used in evaluating chemotherapeutic tumor response in adenocancer patients.

Matrix metalloproteinase-9 (MMP-9) can disrupt the major components, extracellular matrix (ECM), and type IV and V collagen and gelatin; and for this reason, their activity is closely related to the invasion and metastatic ability of tumor cells. Increased expression of matrix metalloproteinases (MMPs) constitutes the ability to digest tumor cells, in particular basal membranes covering blood vessels, basic tissue barriers, thereby promoting cell motility. The tissue inhibitor of metalloproteinase-1 (TIMP1) plays a negative role in the invasion and metastasis of tumor cells by forming a 1 : 1 complex with MMP-9 and inhibiting its enzymatic activity [1–7].

Zhang et al. [17] reported that serum MMP-9 levels correlated with tumor invasion in patients with gastric adenocarcinoma. Shan et al. [18] examined the association of HER-2 gene signal with MMP-9 activity in cells from gastric mucosa and reported that MMP-9 increased via HER2 in gastric cancer pathogenesis. Zhang et al. [19], in their study in patients with gastric adenocarcinoma, reported that the levels of many proteins of the metalloprotease family, including MMP-9, and TIMP1 were significantly higher than those in healthy humans, regardless of histological type.

The main objective of our study was to determine significant differences between the groups in whom curative surgery could be performed and could not be performed (pathological stage > 3) by measuring preoperative serum CK18, MMP-9, and TIMP1 levels in gastric adenocancer patients considered to be operable (clinical stage < 4). In the cases of gastric adenocancer, preoperative diagnostic methods considerably determine the operability in patients; however, the laparotomies performed with the hope of resection cannot reach their goals in a considerable number of patients. In some studies, this rate is over 30% [20]. The rate of the palliative operation group to all cases in our study was 12%.

The serum averages in the palliative operation group were found to be higher than those in the curative group for all three markers, but as a result of the statistical analysis of these markers, no cut-off point could be predicted, which would suggest that the case was inoperable. As a result of the curve fitting analysis, the maximum effect of markers was determined for over 7.7 (the number of metastatic lymph nodes resected in the operation) (Table 5). When CK18 > 5.6 ng/ml, the probability of the number of metastatic lymph nodes being 8 and over increased by 18 times in 95% confidence interval. When MMP-9 > 1528 ng/l, the probability of the number of metastatic lymph nodes being 8 and over increased by 7 times in 95% confidence interval. Similarly, when TIMP1 > 495.7 pg/ml, we found that the probability of the number of metastatic lymph nodes being 8 and over increased by 7 times (Table 6). Although these analyzes were not related to the T-phase and did not predict inoperability, they showed that the N stage was over 3.

Computed tomography, in addition to other imaging techniques, remains the gold standard for the preoperative evaluation of cancer, although it may significantly underestimate the real tumor extension. The present study was designed to confirm the earlier data in a new prospective evaluation, to assess biomarkers, and to incorporate their results into the preoperative evaluation of cancer to help imaging modalities. The addition of the easy and inexpensive biomarkers is capable of correcting this underestimation and helps to decide whether to completely rely on computed tomography or order additional clinical investigations [21]. It is not easy to comment on the superiority of solely computed tomography in an era of rising neoadjuvant oncologic tailor-made treatment for cancer, which combines information of such biomarkers and imaging modalities, especially in the future. In our study, even though the end result is that N3 lymph nodes can be preliminarily detected, we think that these markers can be used as support for imaging methods, but of course not alone, since new work in this area can give more clear information in the future to decrease the rate of shift of cancer stage.

## 6. Conclusion

Although serum CK18, MMP-9, and TIMP1 preop measurements in patients scheduled for curative surgery due to gastric adenocarcinoma did not help to gain any idea of tumor resectability, we concluded that our study had valuable results in significantly predicting the N3 stage, and we believe that studies should be conducted with a larger number of patient groups in this area.

## Figures and Tables

**Table 1:** Patient characteristics.

Age, ave. ± SD (min-max)		64.0 ± 12.2 (42–88)

Age, *n* (%)	<50-year-old	7 (14.0)
	50-70-year-old	26 (52.0)
	>70-year-old	17 (34.0)

Gender, *n* (%)	Male	34 (68.0)
	Female	16 (32.0)

Operation, *n* (%)	R0 resection	44 (88.0)
	Subtotal gastrectomy	24 (48.0)
	Total gastrectomy	20 (40.0)
	Palliative	6 (12.0)

Pathology, *n* (%)	Adenocancer	50 (100)

Tumor A, ave. ± SD (min-max)		34.1 ± 6.7 (21–47)

Clinical T, *n* (%)	3	40 (80.0)
	4	10 (20.0)

Clinical N, *n* (%)	Positive	19 (38.0)

Clinical stage, *n* (%)	2	30 (60.0)
	3	20 (40.0)

Pathological N, ave. ± SD (min-max)		7.5 ± 5.4 (1–18)

Total lymph node, ave. ± SD (min-max)		15.4 ± 6.2 (0–24)

Pathological stage, *n* (%)	2B	7 (14.0)
	3A	12 (24.0)
	3B	14 (28.0)
	3C	11 (22.0)
	4	6 (12.0)

Wound infection, *n* (%)		9 (18.0)
Anastomose leak, *n* (%)		9 (18.0)
Ileus, *n* (%)		4 (8.0)
Atelectasis, *n* (%)		4 (8.0)
Mortality, *n* (%)		14 (28.0)

**Table 2:** CK18, MMP-9, and TIMP1 averages of the patient group.

	Mean ± SD (min-max)
CK18 (ng/ml)	6.0 ± 6.4 (2–29)
MMP-9 (ng/l)	1542.1 ± 2347.7 (129–8237)
TIMP1 (pg/ml)	511.2 ± 452.4 (183–1923)

**Table 3:** Relationship between biomarker levels and tumor size, number of pathological lymph nodes, and pathological stage.

	CK18		MMP-9		TIMP1	
	Rho	*P*	Rho	*P*	Rho	*P*
Tumor size	−0.093	0.521	−0.052	0.718	−0.019	0.897
Pathological N	0.491	**0.001**	0.396	**0.008**	0.497	**0.001**
Pathological stage	0.278	**0.050**	0.346	**0.014**	0.359	**0.011**

**Table 4:** Relationships of biomarker averages with clinical T, clinical N, and, accordingly, with clinical stage.

		CK18	MMP-9	TIMP1
		Ave. ± SD	Ave. ± SD	Ave. ± SD
Clinical T	3	5.8 ± 6.0	1619.0 ± 2427.7	503.5 ± 453.3
	4	6.5 ± 8.0	1234.2 ± 2082.5	542.1 ± 471.7
	*P*	0.942	0.416	0.482

Clinical N	Available	9.8 ± 9.1	2587.2 ± 2879.9	765.8 ± 615.3
	N/A	3.6 ± 1.3	901.5 ± 1706.0	355.2 ± 202.2
	*P*	**0.045**	**0.008**	**0.022**

Clinical stage	2	3.6 ± 1.3	655.8 ± 1045.9	351.1 ± 204.3
	3	9.5 ± 8.9	2871.5 ± 3073.1	751.4 ± 602.3
	*P*	**0.016**	**0.002**	**0.014**

**Table 5:** Curve fitting analysis.

	Mean	SD	Median
Fit for pathological N with CK18 from CURVEFIT	7.7	3.5	6.3
Fit for pathological N with MMP-9 from CURVEFIT	7.6	2.3	6.4
Fit for pathological N with TIMP1 from CURVEFIT	7.7	3.1	6.1
Fit for CK18 with pathological N from CURVEFIT	5.6	3.4	4.3
Fit for MMP-9 with pathological N from CURVEFIT	1528.1	1002.2	1146.5
Fit for TIMP1 with pathological N from CURVEFIT	495.7	246.5	401.8

**Table 6:** Logistic regression analysis of the probability of the number of metastatic lymph nodes being 8 and over.

	*P*	OR	95% CI for EXP(*B*)	
CK18 > 5.6	0.010	18.200	1.979	167.337
MMP-9 > 1528	0.012	7.111	1.536	32.912
TIMP1 > 495.7	0.012	7.111	1.536	32.912
